# Testosterone and androgen receptor pathway modulation in sepsis: immunometabolic mechanisms and therapeutic implications — a scoping review

**DOI:** 10.3389/fendo.2026.1801055

**Published:** 2026-05-07

**Authors:** Mateusz Szczupak, Jacek Kobak, Mateusz Kreczko, Jolanta Wierzchowska, Sabina Krupa-Nurcek

**Affiliations:** 1 Department of Anesthesiology and Intensive Therapy, Copernicus Hospital in Gdańsk, Gdańsk, Poland; 2 Department of Otolaryngology, Faculty of Medicine, Medical University of Gdańsk, Gdańsk, Poland; 3 Department of Otolaryngology, University Clinical Center, Gdańsk, Poland; 4 Department of Anesthesiology and Intensive Therapy, Faculty of Medicine, Medical University of Gdańsk, Gdańsk, Poland; 5 Department of Anesthesiology and Intensive Therapy, University Clinical Center, Gdańsk, Poland; 6 Department of Surgery, Faculty of Medicine, Collegium Medicum, University of Rzeszów, Rzeszów, Poland

**Keywords:** androgen receptor, critical illness, hormonal dysregulation, immunometabolism, intensive care, sepsis, septic shock, testosterone

## Abstract

**Introduction:**

Sepsis remains a major challenge in intensive care medicine, characterized by a dysregulated host response to infection and high mortality. Increasing evidence highlights complex interactions among the endocrine, immune, and metabolic systems, including a potential role of testosterone in modulating the immunometabolic response.

**Aim:**

To systematically map and synthesize current evidence on the role of testosterone as a potential modulator of the immunometabolic response in sepsis.

**Materials and methods:**

A scoping review was conducted in accordance with the Joanna Briggs Institute methodology and PRISMA-ScR guidelines. PubMed, Scopus, Web of Science, Cochrane Library, and EBSCO were searched for studies published between 2015 and 2025. Eligible studies included adult patients with sepsis or septic shock and investigated testosterone levels, androgen receptor activity, or androgen-based interventions. All study designs, including case reports, were considered.

**Results:**

Thirteen studies met the inclusion criteria. The majority of studies reported lower testosterone levels in patients with higher disease severity, reflected by higher SOFA and APACHE II scores, and in non-survivors compared with survivors. Experimental and translational studies have demonstrated that the androgen receptor (AR) pathway regulates cytokine production and immune cell metabolism. Clinical studies evaluating testosterone supplementation reported changes in selected metabolic and clinical parameters; however, no statistically significant improvement in survival was observed.

**Conclusion:**

Testosterone and the androgen receptor pathway may contribute to immunometabolic dysregulation in sepsis. Testosterone deficiency is associated with greater disease severity and increased mortality; however, current evidence does not support the routine use of androgen therapy. Further well-designed translational and clinical studies, incorporating sex-specific analyses, baseline hormonal status, and multi-omics approaches, are required to enable personalized hormonal interventions in sepsis.

## Graphical abstract

**Figure:**
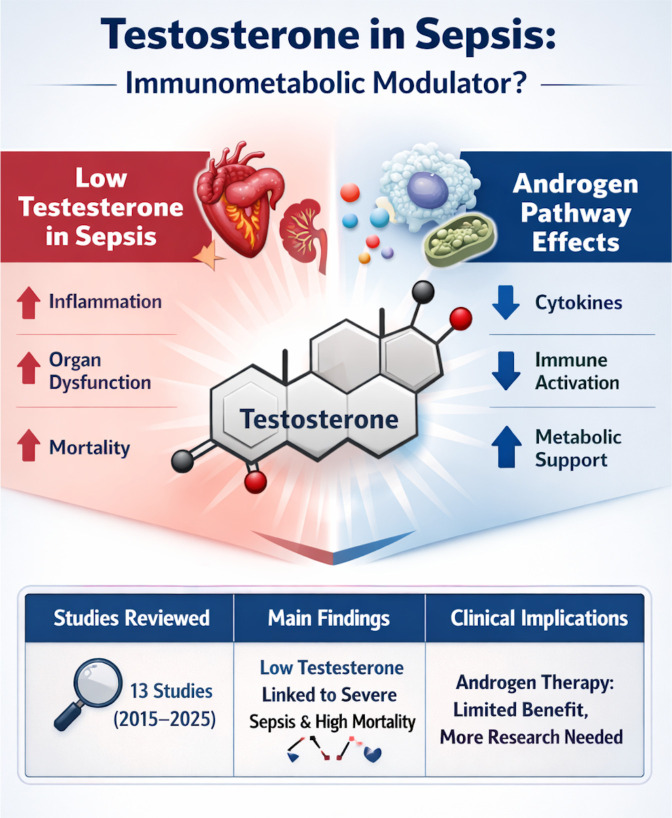

## 1 Introduction

Sepsis remains one of the major challenges in intensive care—it is a complex syndrome of dysregulated host response to infection, leading to organ dysfunction and high mortality. The pathophysiology of sepsis encompasses not only disturbances of the classical inflammatory response but also dynamic changes in the metabolic programs that govern immune cell activation, differentiation, and effector function. This bidirectional relationship between cellular metabolism and immune response is referred to as immunometabolism and includes processes such as shifts between glycolysis and oxidative phosphorylation, alterations in mitochondrial function, and metabolite-driven regulation of inflammatory signaling (1, 2). Throughout this review, the term “immunometabolism” is used to describe the bidirectional interaction between metabolic pathways and immune cell function and is not used interchangeably with immunomodulation alone.

The role of sex hormones in modulating immune responses has become an important area of investigation. In sepsis, documented sexual dimorphism in incidence, severity, and outcomes suggests that sex hormones may influence the trajectory of the host response to infection. Experimental studies and narrative reviews indicate that androgen receptors are expressed in multiple immune cell populations and that androgens modulate neutrophil, macrophage, and T-cell function as well as cytokine synthesis (3, 4).

Testosterone—the primary androgen in humans—exerts pleiotropic effects on immune regulation and metabolism. However, in the context of critical illness, including sepsis, its role remains incompletely understood. It is unclear whether the observed decline in testosterone represents an adaptive response to systemic stress or a maladaptive process contributing to immune dysregulation and worse clinical outcomes (4, 5).

Over the past decade, evidence linking endocrine disruption in sepsis with metabolic dysfunction and clinical outcomes has been growing. Reduced androgen concentrations have been associated with increased inflammation and poorer prognosis in selected cohorts. However, there are no conclusive randomized clinical trials directly evaluating androgen modulation as a therapeutic strategy in sepsis, resulting in a significant clinical gap (6, 7).

Although sepsis is accompanied by multidirectional disturbances across several endocrine axes, including the hypothalamic–pituitary–adrenal, thyroid, somatotropic, and gonadal systems, we deliberately focused this scoping review on testosterone and androgen receptor signaling for three reasons. First, testosterone represents one of the most consistently altered anabolic hormones during critical illness, and its decline has repeatedly been associated with the severity of organ dysfunction, catabolic burden, and poor recovery. Second, compared with other endocrine pathways, testosterone offers a unique bridge between endocrine dysregulation and immunometabolism because androgen receptor signaling directly affects cytokine transcription, innate and adaptive immune cell programming, mitochondrial function, and substrate utilization. Third, testosterone is not only a biomarker candidate but also a potentially actionable pathway, as both androgen replacement and selective androgen receptor modulation can be conceptually examined as therapeutic strategies. Thus, the present review does not aim to diminish the relevance of other hormonal axes in sepsis; rather, it intentionally narrows the scope to one biologically coherent and translationally promising pathway within the broader endocrine response to critical illness (8–10).

In the immunometabolic context, it is important to consider how testosterone may influence immune cell metabolism. Integration of molecular (androgen receptors, intracellular signaling), phenotypic (cellular function, cytokine profile), and clinical (biomarkers, outcomes) data is necessary to determine whether androgens can act as a targeted modulator of the immunometabolic response in sepsis—or whether the observed associations are merely epiphenomena of severe disease (2, 8).

Accumulating evidence indicates that sepsis is a heterogeneous clinical syndrome encompassing distinct biological and metabolic phenotypes. The limited value of isolated interpretation of classic inflammatory markers and the need to integrate them with clinical data are emphasized. Retrospective studies have demonstrated that selected immunological biomarkers differentiate sepsis caused by Gram-positive and Gram-negative pathogens, suggesting differential activation of the immune and metabolic axes in ICU patients (11).

At the same time, attention is drawn to the importance of detailed assessment of organ dysfunction, including reporting individual components of the SOFA scale, which may better reflect the biological heterogeneity of sepsis than the summary score (12). These observations are complemented by data indicating that dynamic hemodynamic parameters correlate better with the current state of perfusion and tissue metabolism than classical static indicators (13). Collectively, these observations support the concept of sepsis as a multi-axial disorder in which the immune, metabolic, and endocrine systems interact closely.

**Figure 1:**
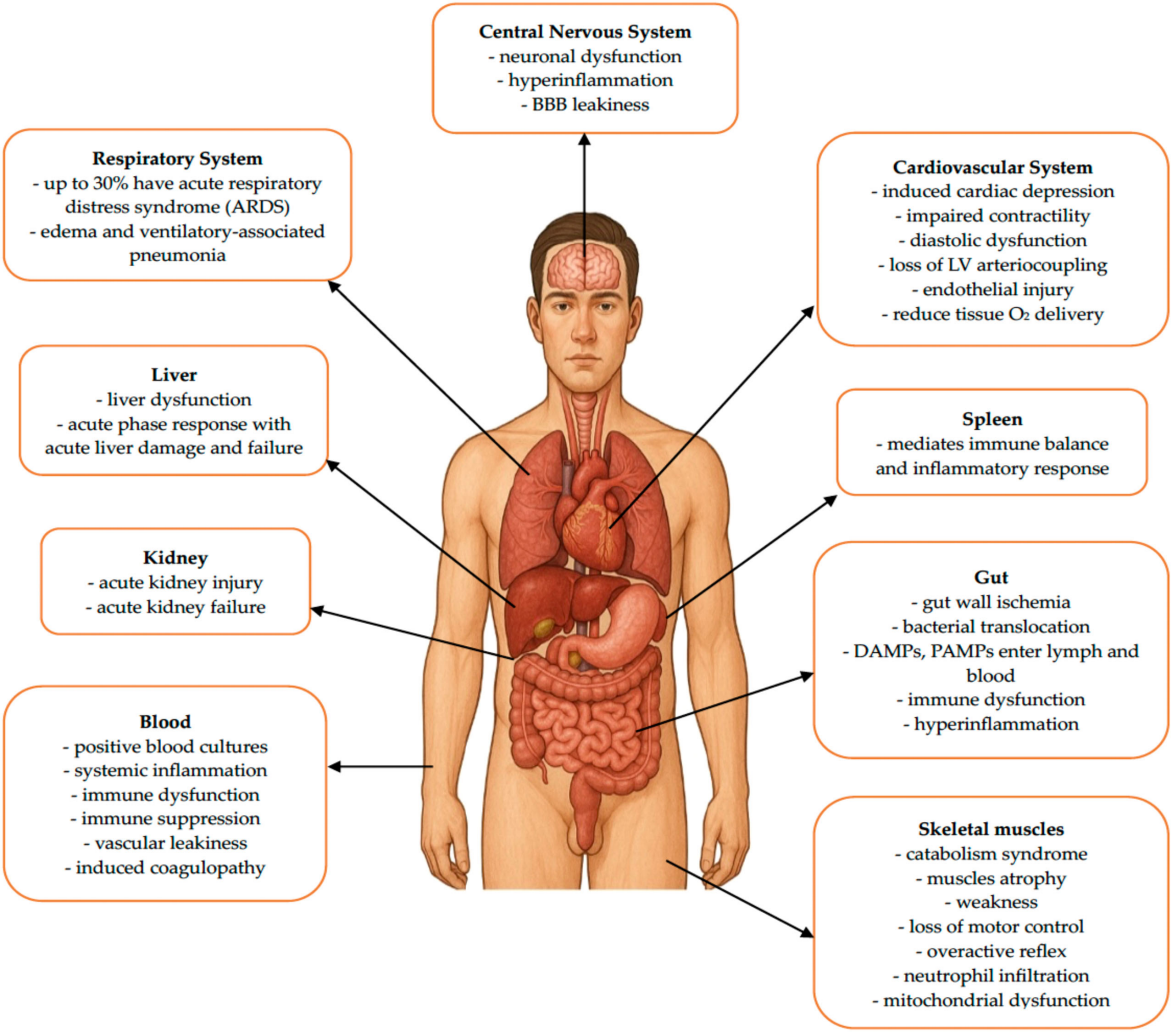
The impact of sepsis on individual systems and organs. LV, left ventricle; O_2_, oxygen; DAMPs, damage-associated molecular patterns; PAMPs, pathogen-associated molecular patterns.

**Figure 2:**
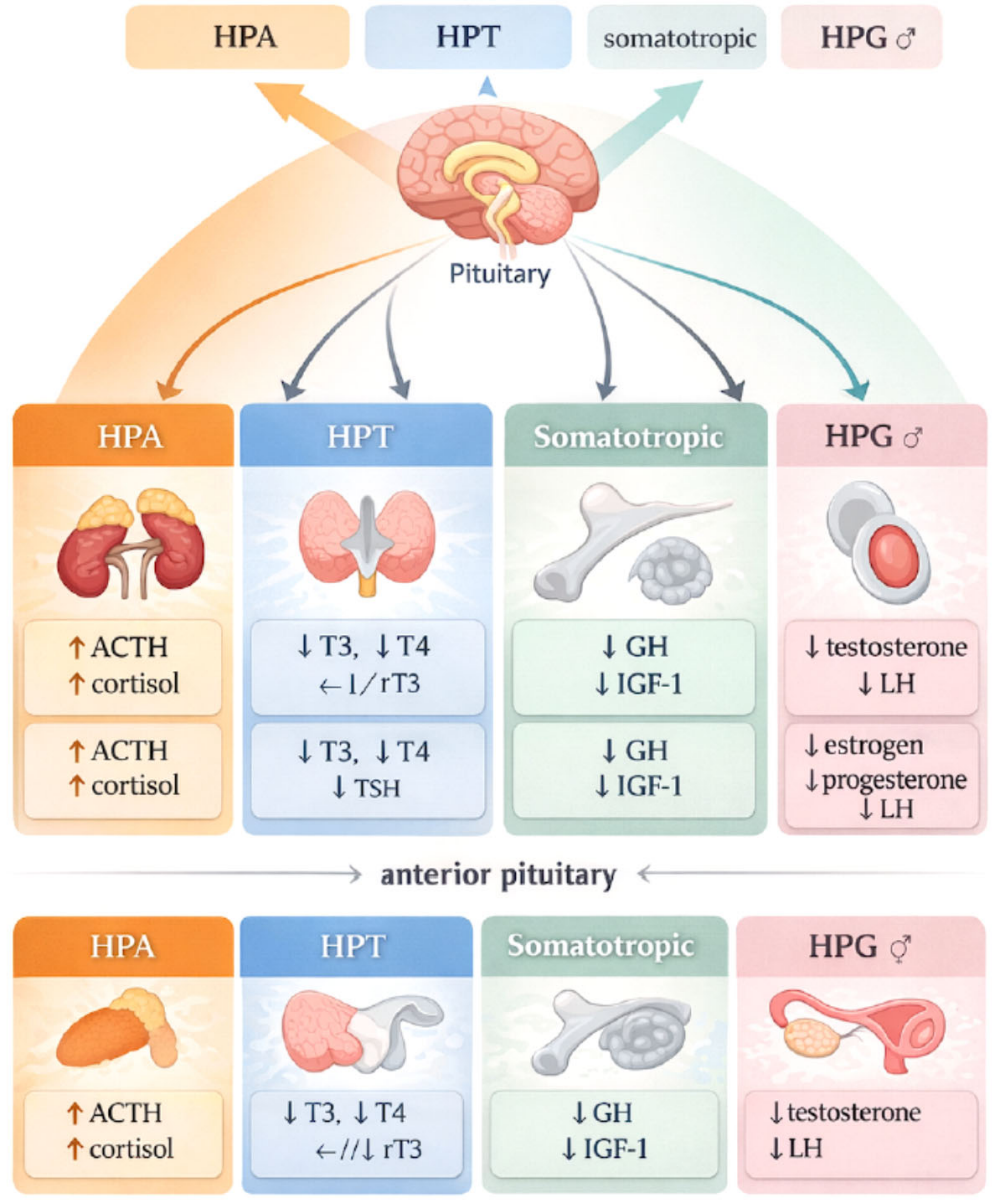
The impact of sepsis on the endocrine system and hormone secretion (9). Symbols: T - increase; 1 - decrease, S - normal, O’-male, f -female. ACTH, adrenocorticotropic hormone (corticotropin); GH, growth hormone (somatropin); GnRH, gonadotropin-releasing hormone (gonadoliberin); HPA, hypothalamic-pituitary-adrenal; HPG, hypothalamic-pituitary-gonadal; HPT, hypothalamic-pituitary-thyroid; IGF-1, insulin-like growth factor I; LH, luteinizing hormone; rT3, reverse triodothyronine; T3, triodothyronine; T4, thyroxine; TSH, thyrotropin.

The aim of this scoping review was to comprehensively map and critically analyze the current evidence regarding the role of testosterone as a potential modulator of the immunometabolic response in sepsis. The review aimed to determine the extent to which available experimental and clinical data support the hypothesis that androgens—through their effects on androgen receptor activity, cytokine profile, and regulation of immune cell metabolism—may shape the clinical course and prognosis of patients with sepsis or septic shock. **Figures 1** and **2** schematically illustrate the impact of sepsis on key systems and organs.

## 2 Materials and methods

### 2.1 Study design

We chose a scoping review approach because our goal was to map and synthesize key concepts related to the use of testosterone in the treatment of sepsis and septic shock. Scoping reviews are a relatively new methodological approach. Currently, there are limited recommendations for choosing between a systematic review and a scoping review when synthesizing evidence, particularly when the literature is not yet comprehensive, is large, heterogeneous, or complex, and therefore not suitable for a more detailed systematic review (14). Our scoping review was prepared according to the methodological framework described in the Joanna Briggs Institute manual for scoping reviews and based on the Preferred Reporting Items for Systematic Reviews and Meta-Analyses extension for Scoping Reviews (PRISMA-ScR) guidelines (15, 16).

This scoping review was conducted in accordance with the PRISMA 2020 guidelines and the PRISMA extension for Scoping Reviews (PRISMA-ScR). The completed PRISMA-ScR checklist is provided as **Supplementary Material** (**Supplementary File S1**).

### 2.2 Inclusion and exclusion criteria

To identify key aspects related to the use of testosterone in the treatment of sepsis and septic shock, we developed a research question that clearly defined the population, concept, and context of the review scope.

To enhance conceptual clarity, the review question was explicitly structured according to the Population–Concept–Context (PCC) framework.

Population (P): adult patients with sepsis and/or septic shock treated in intensive care units.
Concept (C): testosterone levels, androgen receptor (AR) signaling, and their role as biomarkers or therapeutic modulators of the immunometabolic response.
Context (C): critical illness, with a particular focus on sepsis-related endocrine–immune–metabolic interactions.

The primary review question was: What is the role of testosterone and androgen receptor signaling in modulating the immunometabolic response in patients with sepsis or septic shock?

Secondary questions included: 1. Is low testosterone associated with disease severity and clinical outcomes?

2. What mechanistic evidence supports androgen-mediated immunometabolic regulation? 3. Is there clinical evidence supporting testosterone or AR-targeted interventions in sepsis?

Inclusion criteria were as follows:

- Articles published between 2015 and 2025;
- Articles from all study types;
- Articles with full-text access;
- Articles in English;
- Adult patients hospitalized in intensive care units;
- Use of testosterone in the treatment of sepsis and septic shock.

Exclusion criteria included:

- Publications older than 10 years;
- No full-text article;
- Articles in a language other than English;
- Interventions with outcomes unrelated to testosterone, sepsis, and septic shock.

The 2015–2025 time window was selected intentionally to capture the contemporary evidence base most relevant to present-day sepsis definitions, ICU practice, and translational immunometabolic research. This period encompasses the post-Sepsis-3 era, the expansion of omics-based sepsis phenotyping, and the more recent literature on androgen receptor signaling and endocrine–immune interactions in critical illness. Earlier studies remain historically important for understanding gonadal dysfunction in critical illness; however, the purpose of this scoping review was to map current mechanistic and clinically translatable evidence rather than to provide a historical review of endocrine alterations in sepsis (8, 10, 17).

Population

The literature review included studies and reports assessing the efficacy and safety of testosterone as adjunctive therapy in patients with sepsis and/or septic shock. According to the Sepsis-3 definition, sepsis is a life-threatening syndrome of organ dysfunction caused by an abnormal, dysregulated host response to infection. Septic shock is the most severe form of sepsis and is characterized by persistent hypotension requiring vasopressors to maintain a mean arterial pressure ≥ 65 mm Hg and a serum lactate concentration > 2 mmol/L, despite adequate hydration (17). Both conditions are associated with high mortality and a significant incidence of metabolic and hormonal complications (9).

Endogenous testosterone plays a crucial role in these disorders. This hormone regulates numerous physiological processes, including protein synthesis, maintenance of muscle mass, metabolic balance, inflammatory response, and immune system function (18). A decrease in testosterone levels is often observed in sepsis, resulting from inhibition of the hypothalamic-pituitary-gonadal (HPG) axis, oxidative stress, and the action of proinflammatory cytokines (19).

The clinical and experimental studies analyzed focused on assessing the effects of exogenous testosterone on hemodynamic parameters, immunometabolic response, hormonal profile, and mortality in a population of critically ill patients. Attention was paid to the potential role of testosterone in modulating the inflammatory response, improving tissue perfusion, reducing muscle catabolism, and supporting mitochondrial and endocrine functions. Studies describing the association between hypogonadism acquired during sepsis and poorer prognosis and longer intensive care unit stays were also included.

The aim of this review was to determine whether testosterone supplementation can be a reasonable and safe therapeutic strategy in patients with sepsis and/or septic shock, and what the current directions of research are on its use as a potential modulator of the immunometabolic response in critical illness.

Concept

The focus was on assessing the effectiveness of testosterone as adjunctive therapy in patients with sepsis and/or septic shock. The aim of the review was to analyze the impact of testosterone supplementation on clinical outcomes, including improved hemodynamic parameters, reduced inflammation, enhanced protein metabolism, and improved muscle function. The potential impact of testosterone on reducing the risk of multiple organ failure, duration of mechanical ventilation, length of hospitalization, and mortality in the critically ill population was also assessed.

Context

The studies included in the review included patients in whom the effectiveness of testosterone supplementation on the clinical course of sepsis and/or septic shock was assessed.

Types of studies

This review included all available types of studies, including case reports, that assessed the efficacy and safety of testosterone as adjunctive therapy in the treatment of sepsis and/or septic shock. Case reports were included primarily for hypothesis generation and mechanistic insight, rather than for drawing clinical conclusions, given their inherent high risk of bias and limited external validity.

### 2.3 Search strategy

Two authors independently conducted a systematic search of PubMed, Scopus, Web of Science, Cochrane Library, and EBSCO to identify studies published between January 1, 2015, and November 10, 2025. The search strategy incorporated both controlled vocabulary terms and free-text keywords related to sepsis, septic shock, testosterone, androgens, androgen receptor signaling, and hormonal supplementation. Boolean operators (AND/OR) were applied to tailor the search syntax to each database. The full search strategy for PubMed, including all Boolean operators, Medical Subject Headings (MeSH), and field tags, is provided in **Supplementary File S2** to ensure transparency, methodological rigor, and reproducibility of the search process.

All retrieved records were exported to a shared screening file, and duplicate citations were removed prior to title and abstract screening. The initial search was conducted on August 20, 2025, and subsequently updated on November 10, 2025, to capture newly indexed studies.

Title and abstract screening was performed independently by the same two reviewers in accordance with predefined eligibility criteria. Full-text articles of potentially relevant studies were then assessed independently. Any disagreements at either stage were resolved through discussion and re-evaluation of the eligibility criteria. In cases where consensus was not initially achieved, the record was reviewed jointly with the full author team until a final agreement was reached.

To minimize selection bias, eligibility criteria were defined *a priori* using the PCC framework, screening was conducted independently, and reasons for exclusion at the full-text stage were documented and incorporated into the PRISMA flow diagram (15, 16, 20). Inter-rater agreement during both title/abstract screening and full-text assessment was quantified using Cohen’s kappa coefficient (κ). A κ value greater than 0.80 was interpreted as indicating substantial to almost perfect agreement. All discrepancies were resolved through consensus.

### 2.4 Extraction of data

A standardized data-charting form based on JBI guidance for scoping reviews was developed prior to extraction and piloted on a small subset of studies to ensure consistency in interpretation across reviewers. Data charting was performed independently by two authors and subsequently cross-checked. Extracted items included: first author, year, country, study design, population characteristics, exposure/intervention, comparator (if applicable), principal outcomes, main findings, and key methodological limitations relevant to interpretation. Any discrepancies in extracted data were resolved through discussion and verification against the original full text. This iterative approach was used to reduce extraction errors and improve methodological transparency (15, 20).

### 2.5 Critical appraisal process

Consistent with JBI guidance, formal risk-of-bias appraisal is not mandatory in scoping reviews, whose primary purpose is to map the extent, nature, and characteristics of available evidence rather than to generate pooled efficacy estimates (15, 20). Nevertheless, to support critical interpretation, we extracted and narratively reported major methodological limitations of the included studies, including retrospective design, small sample size, lack of randomization, indirectness of evidence, and limited external validity. These considerations are summarized in **Table 1** and explicitly incorporated into the Discussion to avoid overinterpretation of low-certainty clinical findings. Although a formal standardized risk-of-bias tool was not applied, studies were qualitatively assessed for key sources of bias, including study design, confounding, selection bias, and indirectness. This appraisal informed the interpretation of findings.

**Table 1:** Summary of included studies evaluating testosterone and androgen receptor signaling in sepsis, including study design, population characteristics, intervention/exposure, main outcomes, conclusions, and key methodological limitations.

Ordinal number	Author and year	Country/study design	Population	Intervention/exposure	Comparator	Main outcomes	Conclusions	Methodological notes/risk of bias	Reference
1	Ma L. et al., 2020	China; retrospective cohort study	61 adult patients with sepsis admitted to ICU	Testosterone propionate 100 mg intramuscularly, administered twice as an adjunct to standard sepsis therapy	Control group without testosterone administration	↑ serum testosterone and albumin, ↓ APACHE II and SOFA scores, shorter duration of mechanical ventilation and ICU stay; no significant difference in 28-day mortality	Testosterone supplementation may improve metabolic and clinical parameters in septic patients but does not significantly affect short-term survival	High risk of bias (retrospective design, non-randomized, single-center)	(21)
2	Bouwman W. et al., 2021	Netherlands; translational/diagnostic-prognostic study	395 patients with sepsis, infection without sepsis, and healthy controls	Androgen receptor (AR) pathway activity assay in peripheral blood leukocytes by qPCR	Control groups (infection without sepsis or healthy volunteers)	AR pathway activity was ↑ in septic patients; higher AR activity was associated with favourable prognosis (lower mortality, faster recovery)	AR pathway activity may serve as a diagnostic and prognostic biomarker in sepsis	Observational study, no intervention; high analytical quality; low laboratory bias	(22)
3	Wang L. et al., 2024	China; prospective observational study	103 male patients with sepsis-induced cardiomyopathy (SIC)	Measurement of serum testosterone and soluble ST2 (sST2) on ICU admission and day 7	Septic patients without SIC/normal testosterone levels	Lower testosterone and higher sST2 levels correlated with ↑ 28-day mortality; testosterone was an independent predictor of death	Low serum testosterone is a potential marker of disease severity and poor outcome in SIC	Moderate risk of bias (single center, limited multivariate hormonal adjustment)	(6)
4	Stevenson R. et al., 2024	South Africa/Narrative clinical review	Critically ill adults	Review of testosterone dynamics in ICU and sepsis	Lack	Testosterone declines during critical illness; uncertain benefit of replacement	Testosterone suppression may be adaptive; supplementation not recommended acutely	Narrative; medium risk of selection bias	(18)
5	Ko R.E. et al., 2023	South Korea/National cohort	Adults with sepsis in ICU	Sex differences (male vs female)	Lack	Male sex associated with higher mortality	Sex hormones may modulate immune response	Administrative data; confounding likely	(23)
6	Rose N. et al., 2025	Germany/Population-based cohort	2.3 mln hospitalizations with sepsis	Biological sex	Lack	Male sex is related to higher mortality across lifespan	Lifelong androgen–immunity interaction	Registry-based; robust statistics	(24)
7	Min S.Y. et al., 2024	South Korea/Narrative review	ICU adults with sepsis/septic shock	Analysis of gender influence on outcomes	Lack	Testosterone implicated in differential immune response	Androgens shape sepsis outcomes; need mechanistic studies	Narrative; low-quality evidence synthesis	(25)
8	Wernly B. et al., 2021	Austria/Retrospective cohort	ICU patients with sepsis	Sex and hormonal differences	Lack	Minimal differences in ICU mortality	Suggests multifactorial determinants	Large cohort; registry bias	(26)
9	Becerra-Díaz M. et al., 2020	USA/Mechanistic review	Myeloid cells (macrophages, monocytes)	Androgen/AR modulation of innate immune signaling	Lack	AR limits IFN-I and NF-κB activation	AR acts as immune suppressor	Mechanistic; strong evidence	(27)
10	Ainslie R.J. et al., 2024	UK/Review	General immune system overview	Androgen effects on immunity	Lack	Androgens regulate TLR signaling	Broad immunomodulatory impact	High-quality narrative review	(28)
11	Allahverdiyeva S. et al., 2024	Turkey/*In vitro* human macrophages	Primary human alveolar macrophages	Testosterone and estradiol exposure	Vehicle control	↓ cytokine response (TNF, IL-6) with T/E2	Both hormones reduce inflammatory activation	Reproducible; *in vitro* limitations	(29)
12	López A.G. et al., 2024	Spain/Host transcriptomic study	Adult sepsis patients	Gene modules incl. hormone-related genes	Lack	Hormonal response clusters affect prognosis	Supports link between endocrine–immune axes	Good design; small sample	(30)
13	Beltrame A. et al., 2022	Italy/Observational immunology	COVID-19 inpatients (as infection model)	Serum testosterone, estradiol	Healthy controls	Low testosterone lead to hyperinflammation, poor outcomes	Relevance to infection-induced immune dysregulation	Moderate bias; non-sepsis model	(31)

AR, androgen receptor; APACHE II, Acute Physiology and Chronic Health Evaluation II; COVID-19, Coronavirus Disease 2019; E2, estradiol; ICU, Intensive Care Unit; IFN-I, type I interferon; IL-6, interleukin 6; NF-κB, nuclear factor kappa-light-chain-enhancer of activated B cells; qPCR, quantitative polymerase chain reaction; SIC, sepsis-induced cardiomyopathy; SOFA, Sequential Organ Failure Assessment; sST2, soluble suppression of tumorigenicity 2; T, testosterone; TLR, Toll-like receptor; TNF, tumor necrosis factor.

### 2.6 Process for including publications in the review

**Figure 3:**
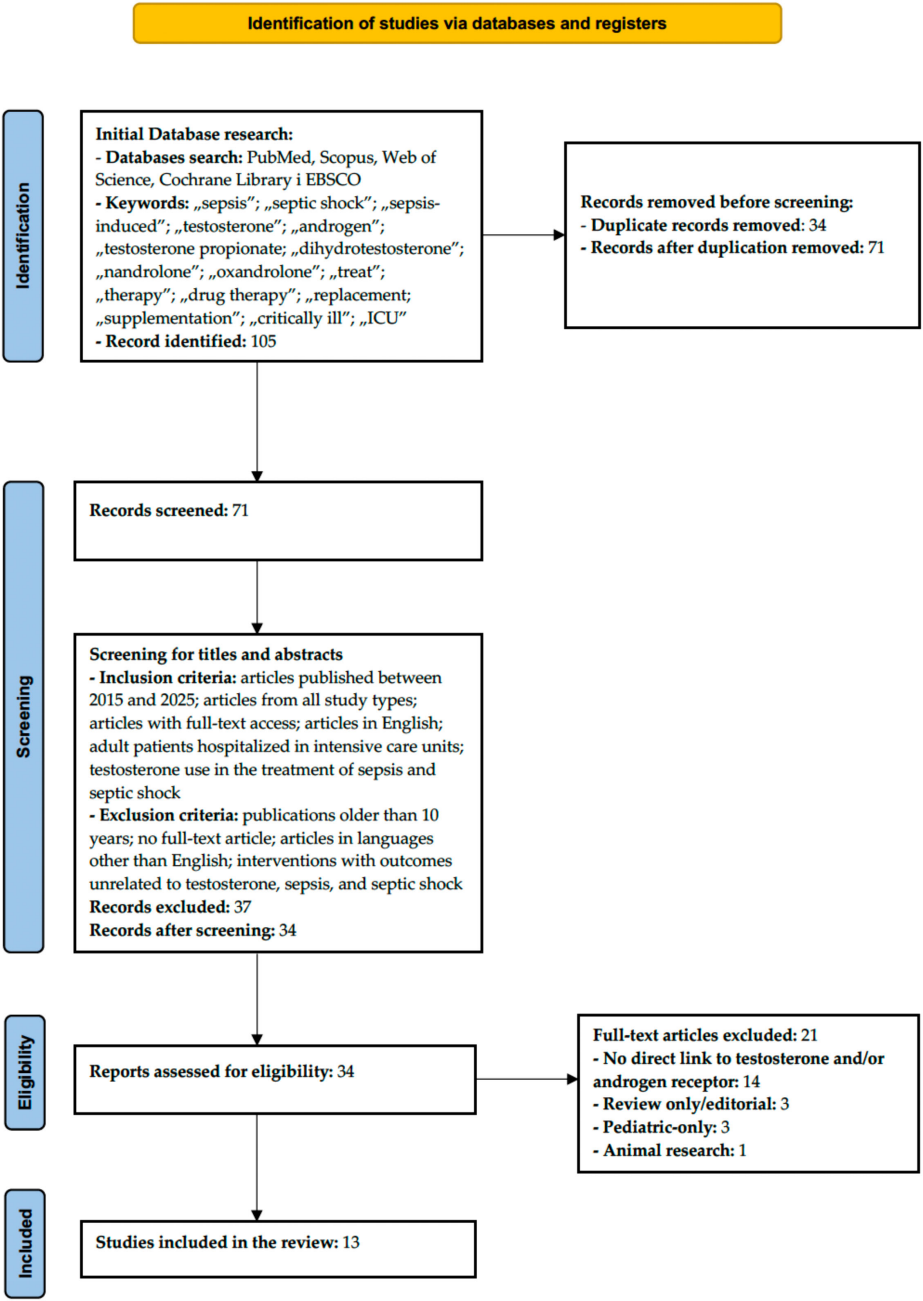
PRISMA flowchart.

The study selection process is presented in **Figure 3** (PRISMA flow diagram), including the number of records identified, screened, excluded (with reasons), and included in the final synthesis.

## 3 Testosterone physiology and its effect on the metabolic system

### 3.1 Testosterone synthesis, regulation, and metabolism

Testosterone is the primary androgen in men, produced by the Leydig cells of the testes. In women, testosterone is produced in the ovaries and adrenal glands in much lower amounts. Testosterone secretion is regulated by the hypothalamic-pituitary-gonadal axis: hypothalamic secretion of gonadotropin-releasing hormone (GnRH) triggers the secretion of luteinizing hormone (LH) and follicle-stimulating hormone (FSH) by the pituitary, which stimulates the Leydig cells in the testes to produce testosterone. Testosterone circulates in the blood primarily bound to proteins (SHBG—sex hormone-binding globulin), with only the free and weakly bound fraction being active. Some testosterone is aromatized to estradiol or reduced to dihydrotestosterone (DHT), which differ in their affinity for the androgen receptor (AR) and tissue activity (18, 32).

Significant disturbances in the HPG axis are observed in states of stress and critical illness. Numerous studies have described decreased total and free testosterone levels in patients with shock, severe trauma, and sepsis. Causative mechanisms include inhibition of the axis by inflammatory cytokines, elevated cortisol levels, altered SHBG expression, and impaired endothelial Leydig cell function. The consequence is a short-term and often prolonged decline in androgen action, which is clinically associated with decreased muscle mass, anabolic metabolic tone, and immune modulation (10, 18).

### 3.2 Molecular mechanisms of testosterone

The mechanisms of testosterone’s action in the immune system include action through the androgen receptor (AR)—a nuclear protein acting as a transcription factor—found in hematopoietic cells, as well as through indirect effects such as modulation of the nuclear factor kappa-light-chain-enhancer of activated B cell (NF-κB) receptor, toll-like receptor (TLR) signaling, nitric oxide (NO) production, and cytokine expression (4, 33). AR is present in both classically androgen-dependent tissues (prostate, muscle) and immune cells (monocytes, macrophages, T and B lymphocytes, dendritic cells) (34, 35). One review paper demonstrated that the androgenic metabolite 3β-androstanediol inhibited NF-κB activation in endothelial cells and reduced the production of proinflammatory cytokines and adhesion molecules (4).

Androgen receptor signaling has also been shown to influence the metabolic programming of Th17 lymphocytes; in men with normal AR activity, Th17 lymphocytes exhibited reduced glutamine metabolism and lower interleukin-17 (IL-17) production (36).

The literature has also shown that AR activity inversely correlates with the expression of genes involved in the immune response in many contexts (e.g., transcriptomic analysis), suggesting that androgen signaling may “suppress” certain proinflammatory programs at the transcriptional level. At the same time, AR blockade in some models increased phagocytic function, indicating a complex, context-dependent effect of AR on immunity (35, 37).

### 3.3 Effects of testosterone on immune cells and cytokines

#### 3.3.1 Neutrophils and macrophages

Preclinical studies and several clinical analyses have demonstrated that androgens modulate innate cell function, influencing chemotaxis, phagocytosis, reactive oxygen species (ROS) production, and the cytokine profile of macrophages. These determinants are complex. In many models, testosterone promotes macrophage polarization toward the M2 (immunoregulatory/repair) phenotype and increases the production of anti-inflammatory cytokines (e.g., interleukin-10) while simultaneously reducing interleukin-6 (IL-6) and tumor necrosis factor alpha (TNF-α) levels. This effect may protect against excessive tissue damage during an acute inflammatory response, but it may also increase susceptibility to chronic infection or immunosuppression (38–40).

#### 3.3.2 Dendritic cells and NK cells

Data suggest that androgens may inhibit dendritic cell maturation and their ability to present antigen. The effect on natural killer (NK) cells is less clear. Both attenuated cytotoxicity and no significant effect have been reported, depending on the experimental model. A reduction in antigen-presenting function may explain some of the observed post-exposure immunosuppression in male cohorts (28, 40).

#### 3.3.3 T lymphocytes

Testosterone has a selective effect on T cell subpopulations. Several studies have shown that androgens inhibit differentiation into Th1 and Th17 lymphocyte lineages and promote an immunomodulatory phenotype (e.g., increased Treg population). The molecular mechanism also involves the regulation of genes encoding phosphatases and the modulation of key transcription factors (e.g., STAT, T-bet, RORγt), resulting in reduced production of interferon gamma (IFN-γ) and IL-17 (34, 41).

#### 3.3.4 B lymphocytes

Data on the effect of testosterone on B lymphocytes most often indicate suppression of humoral activity—lower antibody levels in selected models and conditions—which may partially explain the observed lower susceptibility of men to certain autoimmune reactions, but at the same time potentially weakens antibacterial and antiviral immunity in patients with humoral deficiency (34).

### 3.4 Mechanisms linking androgens to the modulation of inflammation

Androgens influence inflammatory signaling through several important mechanisms: direct transcriptional regulation of pro- and anti-inflammatory genes via the AR; modulation of NF-κB and mitogen-activated protein kinase (MAPK) activity; modulation of pattern recognition receptors; and modification of cellular metabolism, which itself determines the functional phenotypes of immune cells (immunometabolism). A growing body of research indicates that testosterone therapy alters chromatin accessibility at loci associated with JAK-STAT, NF-κB, and other pathways, supporting the concept of androgen-induced epigenetic reprogramming of immune cells by androgens (35, 42).

### 3.5 Effects on metabolism

The immunometabolism of immune cells determines their pro-inflammatory or pro-regenerative phenotype. Testosterone influences metabolism at several levels: it promotes anabolic balance in muscle, improves insulin sensitivity in some cohorts, modulates the expression of glucose transporters (GLUTs) and energy pathway enzymes, and stimulates protein kinase (AMP) signaling in selected tissues. Furthermore, reports indicate that androgens regulate mitochondrial biogenesis and mitochondrial gene expression, which may indirectly influence immune cells’ ability to maintain appropriate metabolic levels during stress. Improving mitochondrial function may limit excessive oxidative stress and modulate the inflammatory response (43–45).

Low testosterone levels are associated with insulin resistance, an unfavorable lipid profile, abdominal obesity, and an increased risk of metabolic syndrome and type 2 diabetes (46, 47). In tissues such as skeletal muscle, liver, and adipose tissue, testosterone deficiency has been shown to reduce GLUT4 expression and glycolytic enzyme levels, which may limit glucose uptake and metabolism (48).

A study on cardiomyocytes showed that testosterone increases glucose uptake and glycolysis by activating protein kinase and the androgen receptor (49).

Testosterone inhibits lipogenesis, increases lipolysis (e.g., by increasing the number of β-adrenergic receptors in adipocytes), and limits the activity of lipoprotein lipase (LPL) in selected adipose tissues (50). In men with hypogonadism and obesity, testosterone therapy was associated with improved mitochondrial function and expression of genes related to lipid metabolism in adipose tissue and reduced fatty liver disease (NAFLD) (51). Furthermore, testosterone deficiency has been shown to promote excessive adipose tissue accumulation (especially visceral), while testosterone treatment or its activation of AR limits this process (animal model studies) (52).

Although data are limited, there is evidence that testosterone may affect mitochondrial biogenesis and oxidative phosphorylation (OxPhos) and reduce oxidative stress. In a study of aging men with low testosterone, increased lipid oxidation was observed after therapy, although there were no significant changes in the expression of genes typically associated with mitochondrial biogenesis (53). In the context of immunometabolism, mitochondrial function plays a key role in the immune response (e.g., macrophage activation), suggesting that testosterone may indirectly modulate the immune system through its effects on cellular metabolism.

## 4 Testosterone and the immune response in sepsis

The last decade has seen a series of studies describing changes in androgen levels in patients with critical illness, including sepsis, and examining the relationship between these changes and inflammatory parameters and clinical outcomes. Most clinical data are observational studies, cohort analyses, and translational work using expression profiles. Numerous preclinical models provide mechanistic data on the effects of androgens on immune cells. Narrative and systematic reviews highlight a consistent picture: critically ill patients experience significant testosterone decline, and lower levels often correlate with more severe disease and poorer outcomes. However, evidence for the effectiveness of testosterone replacement therapy in sepsis is insufficient and fragmented (7, 18).

### 4.1 Sexual dimorphism and hormonal context in sepsis

Sexual dimorphism in sepsis should be interpreted not merely as a demographic observation but as a biologically relevant framework for understanding endocrine–immune interactions. Available evidence suggests that men have a higher incidence of sepsis and, in several cohorts, less favorable outcomes, whereas women may exhibit partially preserved immune resilience, although mortality findings remain heterogeneous across age groups and study designs. Importantly, these differences are unlikely to be explained by testosterone concentrations alone. Rather, they probably reflect the combined effects of sex hormones, sex-chromosome-linked immune regulation, differences in body composition and organ reserve, and potential bias in severity scoring systems and clinical recognition (3, 3, 23, 25).

From a mechanistic perspective, androgen signaling may contribute to sex-specific immune phenotypes by attenuating NF-κB-driven inflammation, modifying Toll-like receptor signaling, and shifting macrophage and T-cell responses toward less proinflammatory states. At the same time, this potentially protective anti-inflammatory effect may become maladaptive during the immunosuppressive phase of sepsis. Conversely, estrogens may preserve endothelial function and immune responsiveness in some contexts. Therefore, sex-related differences in sepsis should be considered dynamic and phase-dependent rather than uniformly beneficial or harmful. This also means that the interpretation of testosterone deficiency in sepsis cannot be separated from biological sex, aromatization, androgen receptor activity, and the broader endocrine milieu (3, 3, 8, 25).

Recent large-scale and registry-based analyses further suggest that sex differences in sepsis outcomes vary across the lifespan and may interact with age-related hormonal changes. Moreover, sex-specific differences in SOFA component values have been reported, raising the possibility that part of the observed disparity may reflect both biological divergence and limitations of uniform diagnostic thresholds. For these reasons, future studies on testosterone in sepsis should not merely adjust for sex as a confounder, but should prospectively stratify patients by sex, hormonal profile, and androgen receptor activity as biologically meaningful variables (24, 24, 26, 54).

### 4.2 Hormonal changes in patients with sepsis

Characteristic gonadal axis disturbances are observed during sepsis and severe systemic illness, including reduced total and free testosterone levels and increased sex hormone-binding globulin and estradiol levels. In male patients with sepsis or other critical illness, it has been found that patients with sepsis requiring mechanical ventilation exhibit significantly reduced total and free testosterone levels already in the first days of hospitalization, with concomitant increases in 17β-estradiol and the estradiol/testosterone ratio. This suggests increased aromatase activity, leading to the conversion of androgens to estrogens (19).

Studies have also shown that the onset of critical illness is associated with a rapid decline in testosterone as early as day 1, typically reaching its nadir around day 3. Return to baseline levels may take weeks or months (9, 18).

The decrease in testosterone in critical illness is explained by several phenomena:

- Proinflammatory cytokines (e.g., IL-6, TNF-α) inhibit Leydig cell function and steroidogenesis, thereby reducing testosterone production (9).
- Increased aromatase activity in peripheral tissues converts androgens to estrogens, explaining the observation of elevated estradiol with low testosterone (9, 19).
- Under conditions of acute critical stress, the hypothalamic-pituitary-gonadal (HPG) axis may be suppressed, leading to decreased pulsatile GnRH secretion, reduced LH/FSH, and, consequently, decreased testosterone secretion (9).
- Possible additional elevation of SHBG concentration, which binds free testosterone, reducing its bioavailability. Although data in sepsis are limited, generally, variable SHBG activity is observed in critical illness (55). Importantly, alterations in sex hormone-binding globulin (SHBG) levels in critical illness may significantly affect the relationship between total and free testosterone. Only a subset of studies accounted for SHBG or albumin levels, limiting the interpretation of biologically active androgen fractions.

The term “critical hypogonadism” appears in the literature as a component of endocrine dysfunction in patients in the intensive care unit (ICU). In this context, critical illness is characterized by multifaceted hormonal dysregulation, encompassing the HPG axis, androgen metabolism, and their effects on anabolism, immunity, and tissue regeneration (18). It is worth emphasizing that although many studies focus on men, gonadal axis dysfunction in sepsis has also been observed in women, including decreased estrogen and progesterone levels because of HPG axis inhibition in critical illness (9).

### 4.3 Correlations between testosterone levels, inflammatory markers, and the patient’s clinical condition

The association between low testosterone levels and the clinical course of sepsis is increasingly well documented, although data specifically on sepsis remain limited. In the study “Testosterone and soluble ST2 as mortality predictive biomarkers in sepsis-induced cardiomyopathy, “ the authors suggested that low testosterone levels correlated with higher APACHE II and SOFA scores and were an independent predictor of 28- and 90-day mortality (6).

In a review by R. Stevenson et al., they indicated that testosterone levels may be correlated with illness severity and treatment outcomes. An important limitation across studies is the heterogeneity in testosterone measurement. Differences in assay methodology (immunoassay vs. mass spectrometry), timing of sampling (early vs. late sepsis), and circadian variability may significantly affect reported concentrations. These methodological inconsistencies limit comparability and interpretation. Furthermore, in the context of overall critical illness, lower testosterone levels were associated with poorer outcomes—higher mortality, prolonged ICU stay, and increased incidence of multi-organ dysfunction (18). Inflammatory biomarkers and clinical parameters in sepsis reflect complex biological phenotypes, and their interpretation requires consideration of the broader metabolic context. Specific biomarker and clinical profiles have been shown to be associated with distinct sepsis and treatment outcomes (11, 56).

Although these studies did not include direct measurements of sex hormones, they provide important interpretive context for the observed associations between low testosterone levels and disease severity. Furthermore, analyses of particularly vulnerable populations, such as elderly patients hospitalized in the ICU, indicate a key role for metabolic disorders and limited physiological reserve as predictors of adverse treatment outcomes (57).

In this context, testosterone can be considered not as an isolated biomarker, but as an element of a complex regulatory network linking the inflammatory response, metabolism, and organ dysfunction. Additionally, studies analyzing the relationships among hormonal axes indicate that the cortisol-to-testosterone ratio or changes in dehydroepiandrosterone/dehydroepiandrosterone sulfate (DHEA/DHEAS) concentrations may have prognostic significance, suggesting that the interrelationships among many hormones (not only testosterone) are important for the course of sepsis (58).

### 4.4 Immunomodulatory mechanisms

Translational molecular studies have demonstrated that activity of the androgen receptor pathway can modulate the immune response in sepsis. Gene expression analyses in patients with sepsis have revealed altered signaling activity of AR-related pathways and correlated expression patterns of genes involved in immunoregulation. Bouwman et al. developed an AR activity index in blood samples, and their work suggests that increased AR pathway activity is associated with immunosuppressive signaling profiles in sepsis, which has diagnostic and potentially therapeutic implications (22).

#### 4.4.1 Inhibiting excessive inflammatory response

Androgens exhibit immunomodulatory properties, including inhibition of excessive proinflammatory cytokine production and modulation of effector cell activity (macrophages, neutrophils, lymphocytes). In animal models, androgen deprivation, such as orchiectomy, enhances the proinflammatory response and worsens outcomes in endotoxemia, whereas restoration of androgen signaling reduces the expression of inflammatory receptors and ameliorates the disease course. The results of these modeling studies suggest a mechanistic basis for the observed correlation between low testosterone levels and the severity of organ damage in sepsis (22, 33).

#### 4.4.2 Support for immune regeneration during the immunosuppression phase

After the initial “cytokine storm, “ sepsis often transitions into a phase of immunosuppression—characterized by lymphocyte anergy, increased immune cell apoptosis, and susceptibility to secondary infections. Testosterone has an anabolic effect and indirectly influences metabolic reserves, which are important for immune regeneration. Furthermore, androgens modulate the proliferation and function of lymphocyte subpopulations and may accelerate the recovery of immune homeostasis. However, these mechanisms are poorly documented in clinical trials in patients with sepsis and require further translational research (8, 33).

#### 4.4.3 Reduction of proinflammatory cytokines and regulation of steroid receptors

There is evidence that androgens reduce the concentrations of selected proinflammatory cytokines (e.g., IL-6, TNF-α) in experimental models and influence glucocorticoid receptor expression, which may modify the response to catecholamines and steroids administered in sepsis therapy. Such effects may translate into tissue protection (less cardiac myocyte damage and less apoptosis) and improved hemodynamic stability (8, 59).

## 5 Potential therapeutic implications

### 5.1 Experimental models

Preclinical studies provide fundamental evidence that modulation of androgen levels influences the inflammatory response and survival in experimental models of sepsis and endotoxemia. In animal models, removal of the androgen source often alters the inflammatory phenotype, leading to an enhanced proinflammatory response and poorer survival. Correcting androgen levels correlates with reduced expression of inflammatory receptors, lower concentrations of certain proinflammatory cytokines, and improved functional parameters (less myocardial damage, better skeletal muscle function) (4, 60).

Furthermore, androgen modulation of the inflammatory response has been described in endotoxemia models – androgens reduced the expression of inflammatory mediators in macrophages, limited tissue apoptosis, and improved survival parameters. Conversely, other studies indicate that dependence on the experimental model, dose, and time of administration suggests that, under certain conditions, androgens may increase the neutrophil inflammatory response and exacerbate tissue damage. This highlights the complex mechanism of action of sex hormones on the immune system (28, 61).

### 5.2 Clinical data

Clinical data regarding the targeted use of testosterone or analogues in critically ill patients are sparse and inconclusive. The literature contains studies on the use of androgen therapy in patients after surgery, trauma, or convalescence from severe illness, suggesting improved anabolic parameters (muscle mass, strength) and reduced inflammatory parameters. There is no evidence of improvement in hard endpoints such as survival or multi-organ failure (18, 62).

Biomarkers and correlation studies are available around ​​sepsis—for example, lower testosterone concentrations correlate with sepsis severity (higher APACHE II/SOFA) and increased mortality in cohort studies. Randomized interventional trials of testosterone supplementation in patients with acute sepsis are practically nonexistent or are preliminary. Clinical trials evaluating the use of testosterone in critically ill patients are ongoing (e.g., clinical studies such as TESTO-TRIAL), but there are currently no multicenter publications or randomized trials confirming its efficacy and safety in the context of sepsis (6, 63).

In practice, clinical conclusions are based primarily on: (1) observational studies (the association between low testosterone and poorer outcomes), (2) experience with the treatment of hypogonadism in community populations (which provide data on long-term safety), and (3) previous rehabilitation studies in post-ICU patients (where anabolic therapy has been considered to accelerate the recovery of muscle mass and function) (61).

### 5.3 Risks, safety concerns, and limitations of androgen therapy

Any discussion of testosterone supplementation in sepsis must be accompanied by a clear statement that current evidence is insufficient to support routine or empirical off-label androgen use in critically ill patients. The endocrine and inflammatory milieu of sepsis is unstable, and extrapolation from outpatient hypogonadism management to acute ICU populations is methodologically unjustified and potentially unsafe. In this setting, testosterone should be considered an investigational pathway rather than a treatment ready for clinical implementation (18, 63–66).

Several safety concerns are particularly relevant. First, testosterone therapy may induce erythrocytosis, alter blood viscosity, and complicate the interpretation of hemoglobin and hematocrit trends in patients already exposed to shock, vasopressors, transfusions, and fluctuating intravascular volume. Second, concerns remain regarding cardiovascular and thromboembolic safety, especially in patients with recent major adverse cardiovascular events, thrombophilia, or profound immobilization. Although data from non-ICU populations are mixed and not directly transferable to sepsis, available endocrine guidance recommends caution in individuals at increased cardiovascular or thrombotic risk (64, 65, 67–69).

Third, testosterone therapy may interact with prostate-related risk stratification, liver function, and concurrent ICU pharmacotherapy. In septic patients, additional uncertainty arises from altered sex hormone-binding globulin concentrations, impaired hepatic metabolism, renal dysfunction, capillary leak, and the absence of validated therapeutic targets for androgen correction during acute illness. These factors make dose selection, route of administration, and treatment monitoring highly uncertain (10, 18, 70).

Finally, there is a conceptual safety concern: if low testosterone during sepsis is partly an adaptive response to systemic stress, indiscriminate supplementation could be biologically inappropriate and might worsen immune dysregulation or prolong immunosuppression in selected patients. For this reason, androgen-based interventions in sepsis should be restricted to rigorously designed clinical studies with predefined eligibility criteria, endocrine monitoring, and prespecified safety outcomes, rather than used off-label in routine ICU practice (18, 18, 60, 61, 64).

### 5.4 Directions of therapy development

1. Need for translational research and randomized clinical trials. Due to the heterogeneity of preclinical evidence and the limitations of observational studies, well-designed translational studies (analyzing AR signaling in immune cells of patients with sepsis) and randomized, controlled clinical trials are necessary to assess: scored immunological criteria (e.g., cytokine profiles, lymphocyte function), clinical outcomes (not only biomarkers but also mortality, multi-organ failure, and ICU stay), and safety (VTE, cardiovascular events). Stratification (e.g., by sex, age, baseline testosterone level, and immune phenotype) is a key element of research (18).
2. Selective androgen receptor modulators (SARMs). SARMs represent an attractive, potential alternative, as they are designed to achieve tissue-selective anabolic effects while reducing the side effects typical of full AR agonists (e.g., effects on the prostate, erythropoiesis). Recent reviews have described progress in the development of SARMs and their evaluation in muscle-wasting diseases. These same characteristics make them candidates for study in populations recovering from critical illness/convalescence and possibly in sepsis, although specific studies in sepsis have not yet been published. SARM studies would need to examine the effect on immunological parameters and thromboembolic risk in the setting of critical illness (71, 72).
3. Personalized therapies. A “one-size-fits-all” approach to hormonal therapy in sepsis is unlikely to be adopted. A credible strategy is personalized therapy: patient selection based on biological sex, age, hormonal status (total and free testosterone, SHBG), immune phenotype (e.g., predominant hyperinflammatory vs. immunosuppressive profile), hemostasis, and comorbidities. This approach would minimize risk and increase the likelihood of identifying a group of patients who will benefit from androgen therapy (24, 62).
4. Therapeutic combinations and intervention timeframe. Defining the “therapeutic window” is crucial—whether androgens should be administered early (to inhibit excessive cytokine levels), late (during the convalescence phase and restoration of anabolic processes), or in specific sepsis phenotypes. Studies may also consider combinations (e.g., androgens + targeted immunostimulation) to minimize the risk of infections and maximize tissue regeneration (4, 18).

## 6 Discussion

This review synthesizes evidence indicating that testosterone and androgen receptor signaling are important regulators of the immunometabolic response in sepsis. Collectively, studies confirm that critically ill patients often experience significantly decreased testosterone levels (so-called “critical hypogonadism”), which correlates with disease severity and negative clinical outcomes (higher SOFA/APACHE II scores, longer ICU stay, higher mortality) (4, 18). Mechanistic studies indicate that AR is expressed in immune cells, and its activity influences NF-κB signaling, TLRs, and cellular metabolism (22, 27).

A central conceptual question is whether reduced testosterone in sepsis represents an adaptive or maladaptive response. On the one hand, suppression of anabolic and immunomodulatory pathways may limit excessive inflammation and metabolic demand during acute stress. On the other hand, prolonged androgen deficiency may contribute to immunosuppression, muscle catabolism, and delayed recovery. Current evidence does not allow clear differentiation between these paradigms, and the effect is likely phase-dependent.

Our analysis also confirms that androgen effects are context-dependent. In experimental models, androgens can both suppress excessive inflammatory responses (reducing IL-6 and TNF-α and limiting apoptosis) and promote immunosuppression, thereby increasing susceptibility to secondary infections (4, 5). In clinical trials, the therapeutic effect of testosterone supplementation in sepsis remains unclear: small studies suggest improvements in metabolic parameters and some clinical indicators, but randomized, multicenter trials have not confirmed an effect on survival (8, 21).

An important limitation of the current evidence base is that the clinical signal in favor of testosterone supplementation relies predominantly on low-certainty data. In particular, the study by Ma et al. reported improvements in albumin concentration, APACHE II score, SOFA score, duration of mechanical ventilation, and ICU length of stay after testosterone propionate administration; however, this study was retrospective, single-center, non-randomized, and susceptible to confounding by indication and co-interventions. Therefore, its findings should be interpreted as hypothesis-generating rather than practice-changing. More broadly, the available literature is characterized by substantial heterogeneity in population selection, timing of hormone measurement, endocrine endpoints, and clinical outcomes, which markedly limits causal inference. This distinction is crucial: low testosterone may represent a biomarker of disease severity, a maladaptive component of critical illness, or an adaptive response to severe systemic stress, and current studies do not allow these possibilities to be disentangled with confidence (10, 18, 18, 21). Additionally, publication bias cannot be excluded. Studies reporting significant associations between testosterone levels and clinical outcomes may be overrepresented, whereas negative or null findings may be underreported, particularly in small observational cohorts.

A notable contribution of recent research has been the development of tools to estimate androgen receptor pathway activity in peripheral leukocytes. These approaches suggest that AR signaling may help identify biologically distinct sepsis phenotypes and may have diagnostic or prognostic value. In parallel, transcriptomic and metabolomic studies support the existence of complex interactions between sex hormones and host-response programs, potentially enabling future selection of patients most likely to benefit from targeted hormonal interventions (22, 30).

Available data from the last decade indicate a significant role of testosterone in the immunometabolic course of sepsis, but the current evidence base does not allow for the recommendation of routine androgen therapy in septic patients. Randomized clinical trials and comprehensive translational studies are necessary that consider biological sex, hormonal status, and the molecular complexity of sepsis.

This review is subject to several methodological limitations. Only English-language studies were included, which may introduce language bias. The search was restricted to selected electronic databases and did not systematically include gray literature, potentially resulting in an incomplete capture of relevant evidence. Furthermore, the substantial heterogeneity in study designs, populations, and outcome measures precluded quantitative synthesis.

## 7 Research gaps and directions for future research

### 7.1 Lack of studies comparing biological sex in the immunometabolic response to sepsis

There are strong indications that the immune response and metabolism during sepsis exhibit sexual dimorphism—both clinically (differences in course and outcomes) and molecularly (differences in cytokine profiles and metabolic pathway activity). However, most clinical and translational studies either do not report results by sex or are not designed to adequately test sex/intervention interactions. Consequently, reliable, prospective comparisons of immunometabolic phenotypes of sepsis in women and men, with integrated assessment of hormonal status and metabolic markers, are lacking. This gap has direct implications for therapeutic decisions (3, 60).

### 7.2 Insufficient data on the use of testosterone in sepsis

Clinical data regarding the targeted administration of testosterone (or analogs) to patients with sepsis are very limited. Observations linking low testosterone levels with a worsening disease course do not necessarily confirm the efficacy of supplementation; there are no large, randomized trials assessing the safety and efficacy of hormonal therapy in the context of sepsis. Available studies primarily consist of observational studies or experience in postoperative/traumatic patients, often with a disease profile other than sepsis (18, 21).

### 7.3 The need to integrate omics research

Sepsis is a heterogeneous disease at the molecular level. Biomarkers are rarely sufficient for precise patient phenotyping. Integration of multi-omics data—circulating blood transcriptomics, plasma proteomics, serum metabolomics, and epigenomics—will enable: (1) identification of patient subgroups that could benefit from androgen modulation, (2) understanding of androgen-dependent molecular mechanisms, and (3) selection of prognostic and predictive biomarkers of therapeutic response. A growing number of studies have sequenced and analyzed blood transcriptomes from patients with sepsis, demonstrating the utility of metabolomics for stratification; however, few studies combine these approaches with sex hormone measurements and functional analyses of the androgen receptor (73–75).

### 7.4 Interactions of testosterone with other hormones

Sepsis is a state of global hormonal dysregulation; the hypothalamic-pituitary-adrenal axis, insulin, leptin, and other metabolic hormones play crucial roles in determining the course of the disease. Interactions between testosterone and these axes are multifaceted: for example, cortisol can inhibit the HPA axis and lower testosterone; conversely, androgens modify glucose metabolism and insulin sensitivity and affect adipokines (e.g., leptin, adiponectin). In sepsis, these relationships are complicated by impaired hormone transport, inflammation, and treatment (glucocorticoids, insulin therapy). Gaps include the lack of data on: (1) the modulatory effects of testosterone on HPA and cortisol response in sepsis, (2) whether testosterone administration modifies septic insulin resistance and the risk of hyperglycemia, and (3) how adipokines mediate the immunometabolic effects of androgens (8, 76, 77).

### 7.5 Priority future research recommendations

1. Establishment of an international registry and biobank of sepsis patient samples, with mandatory recording of biological sex, sex hormone levels, and a sampling protocol for multi-omics analyses.
2. Randomized trials assessing the safety and efficacy of androgen therapy in a strictly selected population, with the goal of analyzing the sex effect.
3. Development of translational programs combining AR analyses of patients’ immune cells to enable sepsis phenotyping and, consequently, the identification and differentiation of predictive biomarkers.

## 8 Conclusions

Testosterone appears to be a clinically relevant modulator of the immunometabolic response in sepsis. The decline in total and free testosterone observed in critically ill patients correlates with disease severity and adverse outcomes, while androgen receptor signaling may provide additional insight into host-response heterogeneity. However, the currently available evidence is predominantly observational, mechanistic, or indirect, and does not justify routine testosterone supplementation in patients with sepsis or septic shock.

At present, testosterone should be viewed primarily as a candidate biomarker pathway and a hypothesis-generating therapeutic target rather than an established treatment modality. Future research should integrate biological sex, hormonal status, androgen receptor activity, and multi-omic phenotyping to identify clinically meaningful subgroups and determine whether androgen-targeted interventions can be both effective and safe in critical illness.

## Glossary

AR: Androgen Receptor
SOFA: Sequential Organ Failure Assessment
APACHE II: Acute Physiology and Chronic Health Evaluation II
mmHg: Millimeters of mercury
mmol/L: Millimoles per liter
HPG: Hypothalamic–Pituitary–Gonadal Axis
GnRH: Gonadotropin-Releasing Hormone
LH: Luteinizing Hormone
FSH: Follicle-Stimulating Hormone
SHBG: Sex Hormone-Binding Globulin
DHT: Dihydrotestosterone
HPA: Hypothalamic-Pituitary-Adrenal Axis
NF-κB: Nuclear Factor Kappa-Light-Chain-Enhancer of Activated B Cells
TLR: Toll-like Receptor
NO: Nitric Oxide
IL-17: Interleukin 17
ROS: Reactive Oxygen Species
TNF-α: Tumor Necrosis Factor Alpha
NK: Natural Killer
MAPK: Mitogen-activated Protein Kinases
GLUT: Glucose Transporter
GLUT 4: Glucose Transporter type 4
AMPK: AMP-activated protein kinase
IL-6: Interleukin 6
ICU: Intensive Care Unit
DHEA: Dehydroepiandrosterone
DHEAS: Dehydroepiandrosterone sulfate
VTE: Venous Thromboembolism
SARMs: Selective Androgen Receptor Modulators
