# Supplementary material for: Testosterone and androgen receptor pathway modulation in sepsis: immunometabolic mechanisms and therapeutic implications — a scoping review
Source: Front Endocrinol (Lausanne). 2026 May 7;17:1801055. doi: 10.3389/fendo.2026.1801055 (PMC13189809; doi:10.3389/fendo.2026.1801055)
Supplement: Supplementary file 1 [file DataSheet1.pdf]

Supplementary Materials

Supplementary File S1. PRISMA-scR checklist

Section	Item No.	Checklist Item	Reported (Yes/No)	Location in Manuscript
Title	1	Identify the report as a scoping review	Yes	Title page
Abstract	2	Provide a structured summary including background, objectives, eligibility criteria, sources, results, and conclusions	Yes	Abstract
Introduction	3	Describe the rationale for the review	Yes	Introduction (Section 1)
Introduction	4	Provide an explicit statement of objectives/questions	Yes	End of Introduction; Section 2.2
Methods	5	Indicate whether a protocol exists and where it can be accessed	No	Not registered
Methods	6	Specify eligibility criteria	Yes	Section 2.2
Methods	7	Describe all information sources	Yes	Section 2.3
Methods	8	Present full electronic search strategy	Yes	Section 2.3; Supplementary File S2
Methods	9	Describe selection process of sources of evidence	Yes	Section 2.3
Methods	10	Describe data charting process	Yes	Section 2.4
Methods	11	List and define all data items	Yes	Section 2.4
Methods	12	Describe critical appraisal methods (if done)	Yes	Section 2.5
Methods	13	Describe methods for synthesis of results	Yes	Sections 3–6
Results	14	Provide numbers of sources screened, assessed, and included	Yes	Figure 3
Results	15	Describe characteristics of included sources	Yes	Table 1
Results	16	Present critical appraisal within sources	Yes	Table 1; Discussion
Results	17	Present results of individual sources	Yes	Sections 4–5
Results	18	Summarize synthesis of results	Yes	Section 6
Discussion	19	Summarize main results	Yes	Section 6
Discussion	20	Discuss limitations of evidence and review process	Yes	Section 6
Discussion	21	Provide conclusions and implications	Yes	Section 8

Section	Item No.	Checklist Item	Reported (Yes/No)	Location in Manuscript
Funding	22	Describe funding sources	Yes	Funding section

Supplementary File S2. Full PubMed search strategy used in the present scoping review

Element	Description
Database	PubMed (National Library of Medicine)
Platform	PubMed interface (https://pubmed.ncbi.nlm.nih.gov/)
Initial search date	20 August 2025
Search update	10 November 2025
Time frame	1 January 2015 – 10 November 2025
Language restriction	English
Study type restriction	None (all study types included: observational, experimental, reviews, case reports)
Population concept	Sepsis and septic shock
Exposure/Concept	Testosterone, androgens, androgen receptor signaling
Context	Immune response, immunometabolism, inflammation
Search fields	MeSH terms and Title/Abstract fields ([tiab])
Boolean logic	Synonyms combined using OR; concept blocks combined using AND
Truncation	androgen* used to capture lexical variants (androgen, androgens)
Phrase searching	Quotation marks used for exact phrases (e.g., "septic shock", "androgen receptor")
Full search string	(("Sepsis"[Mesh] OR "Shock, Septic"[Mesh] OR sepsis[tiab] OR "septic shock"[tiab]) AND ("Testosterone"[Mesh] OR "Androgens"[Mesh] OR "Receptors, Androgen"[Mesh] OR testosterone[tiab] OR androgen*[tiab] OR "androgen receptor"[tiab] OR "AR signaling"[tiab]) AND ("Inflammation"[Mesh] OR "Immune System"[Mesh] OR "Immunologic Factors"[Mesh] OR "immune response"[tiab] OR immunometabolism[tiab] OR inflammation[tiab])) AND english[la] AND ("2015/01/01"[dp] : "2025/11/10"[dp])
Additional search methods	Manual screening of reference lists of included studies
Deduplication	Duplicate records removed prior to screening
Screening process	Independent title/abstract and full-text screening by two reviewers
Reproducibility	Full search string, filters, and field tags reported to ensure reproducibility
